# The impact of fathers' physical and psychosocial work conditions on attempted and completed suicide among their children

**DOI:** 10.1186/1471-2458-6-77

**Published:** 2006-03-27

**Authors:** Ostry Aleck, Maggi Stefania, Tansey James, Dunn James, Hershler Ruth, Chen Lisa, Louie Amber, Hertzman Clyde

**Affiliations:** 1 Department of Health Care and Epidemiology, University of British Columbia, Vancouver, Canada; 2 University College of the Cariboo-Thompson Rivers University, Kamloops, Canada; 3 Oxford University, Oxford, UK; 4 University of Toronto, Toronto, Canada

## Abstract

**Background:**

Adverse employment experiences, particularly exposure to unemployment and the threat of unemployment, have been strongly associated with several adverse mental and physical health outcomes including suicide. However, virtually no research has been conducted on the trans-generational impact of parental working conditions on attempted or completed suicide among their children.

**Methods:**

We conducted a nested case control study based on a cohort, gathered in the western Canadian province of British Columbia, of male sawmill workers and a second cohort of their children. Physical and psychosocial work conditions to which fathers were exposed during the first 16 years of their children's lives, measured using the demand/control model, were linked to hospital suicide records (attempted and completed) among their children.

**Results:**

Two hundred and fifty children in the cohort attempted or committed suicide between 1985 and 2001. Multivariate models, with partial control for father's mental health outcomes prior to their child's suicide demonstrate, 1) a strong association between low duration of father's employment at a study sawmill and attempted suicide for their male children, 2) elevated odds for attempted suicide among female children of fathers' employed in a sawmill job with low control and, 3) a strong association between fathers in jobs with low psychological demand and completed suicides among male children.

**Conclusion:**

Exposure of fathers to adverse psychosocial work conditions during the first 16 years of their children's life was associated with greater odds for attempted and completed suicide among their children.

## Background

Adverse employment experiences, particularly exposure to unemployment and the threat of unemployment, have been associated with a range of adverse mental and physical health outcomes [1-3] including suicide [4-6]. However, virtually no research has been conducted on the trans-generational impact on the health of their children of adverse parental working conditions.

Exposures to varying socio-economic circumstances, during childhood and beyond, constitutes a unique "life exposure trajectory," which will manifest as different expressions of health and well-being over the life-course [7]. The possible long-term exposure/expression relationships can be conceptualized in three generic models: *latency, cumulative*, and *pathway. Latency *refers to relationships between an exposure at one point in the life course and a health outcome years or decades later, irrespective of intervening experience. *Cumulative *refers to multiple exposures over the life course whose effects on health combine. These may be either multiple exposures to a single recurrent factor (e.g., chronic poverty) or a series of exposures to different factors. Finally, *pathways *are dependent sequences in which an exposure at one stage of the life course influences the probability of other exposures later in the life course, which are the proximate causes of disease expression [7-13].

While this framework is useful to describe the biological mechanism by which the impacts of adverse family socio-economic environments might act to affect the health of children, it tells us little about which dimensions of family socio-economic environment may be most salient.

Research conducted over the past 75 years has shown that parental access to employment (or lack thereof) directly impacts the psychological health, behaviour, and educational outcomes of their children likely through the "sequential effects on parents' job-related affect and parenting behaviours" [14-17].

In order to test the possibility that exposure of parents to adverse employment conditions may impact their children's mental health status, unusual data sets are required which bring together occupational exposure information for the parent(s) and mental health outcome data for their children. Over the past 15 years we have gathered a cohort of approximately 30,000 sawmill workers in British Columbia (BC) and a second cohort consisting of their children with the appropriate data elements in place to conduct this type of study. The purpose of this paper is investigate the impact of fathers' psychosocial and physical work conditions during the first 16 years of their children's life on attempted and completed suicides among their children, during childhood through to middle-adulthood.

Individuals exposed to adverse socio-economic circumstances during childhood tend to be less healthy later in life compared to individuals who experienced a more advantaged childhood. This relationship appears consistent in well-designed longitudinal studies across many physical health outcomes into adulthood (e.g., self-reported health, coronary heart disease, body mass index, and other chronic conditions) [18-24]. These results also hold for a range of psychological and behavioural outcomes [18,25]. And, the way in which such exposures in childhood might manifest in later adverse health outcomes has biological plausibility [7].

In a literature originating in the Great Depression of the 1930s and continuing through to the present, adverse family income and employment circumstances have been linked to negative mental health, behavioural, and physical health outcomes among children. Several studies have shown that major family income losses between 1929 and 1933 increased the emotional instability of fathers but not mothers [26,27]. These studies indicate that male reaction to income loss was increased anger and a tendency for fathers to be punitive and arbitrary when disciplining their children [14,15,28].

After a hiatus of five decades, several American and Canadian studies were conducted on the impacts of family job insecurity on children's mental health outcomes and school-related problems [16,17]. Most of this research identified an association between father's punishing and rejecting behaviours and adverse school behaviours and academic outcomes among their children [29,30]. A number of researchers have further postulated that parental punishing behaviour predicts externalizing behaviours (i.e., anger-based acting out behaviours) in their children whereas parental rejecting behaviours predict internalized symptoms (i.e. depression and anxiety) [31].

Only one study was found that explored the link between the psychosocial work conditions experienced by employed fathers and outcomes among their children. In this Canadian study, conducted among 189 grade 4 and 5 students, utilizing Karasek's demand/control model, Stewart and Barling [17] demonstrated that fathers psychosocial conditions at work influenced their parenting behaviours, which in turn affected their children's' behaviour.

These studies indicate first, that father's work may have a greater influence on children's behaviour and educational and health outcomes than mother's work. Second, they indicate that the potential pathway linking father's work conditions to their children's health may be through altered parenting behaviour, in particular, more disciplining and authoritarian behaviours meted out to children when fathers come under pressure at work.

The present study investigates the impact of fathers' psychosocial and physical work conditions on attempted and completed suicides in a cohort of children residing mainly in rural forest-industry dependent communities in the province of British Columbia in Western Canada.

## Methods

This study is based on a cohort of male sawmill workers for whom we have obtained data on their history of unemployment and physical and psychosocial work conditions. Using birth files from the provincial vital statistics registry, we developed a second cohort consisting of most of their children. This cohort was linked to the BC Linked Health Database (BCLHDB) in order to obtain attempted and completed suicide outcomes. Ethical approval was obtained from the University of British Columbia and the BC Ministry of Health to conduct this study.

### A) The original cohort

The original cohort was gathered in order to conduct an occupational study on the effects of chlorophenol anti-sapstain exposure among BC sawmill workers. We identified 14 sawmills located in BC and accessed personnel records for workers who had worked, for at least one year, in one of these mills anytime between 1950 and 1998. The cohort consists of 28,794 males. Personal identifying information for eligible workers and complete job history records were abstracted from personnel records (See Hertzman et al. [32] for a complete description of methods used to assemble this cohort).

### B) Exposure assessment

From the job history records we obtained the number of episodes of unemployment, job mobility (classified as upward, downward or stable), and occupation (manager, skilled trades, machine operator, or unskilled). We obtained historical estimates of job control, demands, noise, and social support in two ways: 1) 4 experienced job evaluators (two union and two management) in the BC sawmill industry filled out the demand/control questionnaire to obtain a retrospective estimation for all basic job titles prior to 1975 (See Ostry, Marion, & Green et al. [33], for these methods); 2) a panel of senior workers was selected in each mill and filled out Karasek's questionnaire for basic job titles in their mill for two time periods (1975 to 1985), (1985 to 1998) (See Ostry, Marion & Green et al. [34], for these methods). These expert estimates for control, psychological demand, physical demand, social support, and noise were then applied to the job history database in the fathers' cohort.

### C) The children's cohort

The cohort of adult sawmill workers was linked to the BC birth file in order to identify all of the children of these workers born in British Columbia between 1952 and 2000 (see Dimich-Ward et al. [35] for these methods). There were 37, 827 children in the cohort of whom 19,833 satisfied our eligibility criteria (i.e. father had at least one year of work in a study sawmill while their child or children were aged 0 – 16).

### D) The dependent variables

Using probabilistic linkage techniques the children's cohort was linked to the BC Linked Health Database (BCLHDB), consisting of person-specific, longitudinal records on all British Columbians (see Hertzman et al. [32] for these methods. We were able to link 88 percent of the members of the children's cohort to the BCLHDB. These files contain data on deaths, hospital discharges, and other medical encounters for the years 1985 through to 2001. The records are stored separately but have been indexed with an individual service-recipient-specific code so that the records of groups of individuals can be linked across files for specific research projects.

A Data Access Subcommittee consisting of health ministry personnel, staff from the BC Ministry of Information and Privacy, and the Centre for Health Services and Policy Research has been established to handle requests for linkage to the BCLHDB and to ensure that such requests meet scientific and ethical standards, are in the public interest, and conform with the Freedom on Information and Protection of Privacy Act.

Using ICD9 codes available in the hospital discharge database we were able to identify any suicide case (completed and attempted) that occurred between Jan 1985 and March 31st, 2001. A suicide case was defined as anyone with a hospital discharge or death record coded with ICD9 code 950 to 950.9.

### E) Building the exposure file

We determined children's exposure to adverse family socio-economic circumstances by applying father's exposure (in terms of job mobility, unemployment experience, and exposure to control, psychological demand, physical demand, social support, and noise) during each year of the child's life from age zero to the end of their 16th birthday.

### F) Case control analysis

Cases were identified for each completed and attempted suicide. Using survival-time to case-control on STATA 8.0, three controls were selected for each case matched on age and gender. Controls were chosen randomly with replacement from the set at risk. The set at risk were all the children in the cohort, born between 1952 and 1998, whose father worked in a study sawmill for at least one year during the first 0–16 years of the child's life. These could be anyone at risk who also satisfied the matching criteria who had not attempted suicide at the time of diagnosis of the case.

Other socio-demographic characteristics of father's that were available in the cohort database and, potentially of importance in relation to children's suicides, were father's marital status (married, divorced, widowed, or single) and race (East-Indian, Chinese and other). As well, we were able to determine, from the BCLHDB, whether a father had a completed or attempted suicide, mental health diagnosis, or alcohol-related mental health diagnosis, prior to their child's attempted or completed suicide.

Statistical analyses were conducted using conditional logistic regression on STATA 8.0. Univariate models were first run with each independent variable. Multivariate models were developed in three steps. Father's socio-demographic characteristics were controlled for in model 1. In the second step, father's suicide, mental health diagnosis, or alcohol-related mental health diagnosis prior to their child's suicide attempts or completion were controlled. Fathers' occupation at the time prior to their child's suicide was added to the final model.

## Results

Of the 19,833 children in the cohort, 252 attempted or committed suicide between 1985 and 2001 (Table 1). Approximately three quarters of children completing suicide were male whereas approximately two-thirds of children attempting suicide were female. More children of unmarried fathers attempted and completed suicide compared to children of married fathers and, more children of Caucasian fathers attempted or completed suicide compared to children of non-Caucasian fathers (Table 2).

For male and female children, univariate results show that father's marital and ethnic status was not associated with attempted suicide. For males, father's prior diagnosis for an alcohol-related mental health condition was associated with elevated odds for their child's attempted suicide. For females, father's prior diagnosis for a mental health condition was associated with elevated odds for attempted suicide (Table 3).

Additionally, for males, father's employment in an unskilled sawmill job prior to their child's suicide was associated with increased odds for their child's attempted suicide. Lower duration of employment at a sawmill was also associated with elevated odds for attempted suicide for male children. Univariate analyses demonstrated that fathers' lower duration of employment at a sawmill, low control and high physical demand were also associated with elevated odds for attempted suicide among female children. Univariate analyses also demonstrated that male children of tradesmen had significantly lower odds for attempted suicide relative to other occupations (Table 3).

As only 4 female children completed suicide univariate models were not run for female children. For male children, univariate models demonstrated no association between father's socio-demographic status and completed suicide. Further, univariate models showed association with completed suicide except for low psychological demand which was associated with elevated odds for completed suicide (OR = 0.46; CI = 0.26–0.79; p = 0.00).

Fully controlled multivariate models demonstrate a strong association between low duration of father's employment at a study sawmill and attempted suicide for male children. (Table 4). Fully controlled multivariate models demonstrate that elevated odds for attempted suicide among female children is associated with prior mental health diagnosis for their fathers and employment in a sawmill job with low control (Table 5). Finally, fully controlled models indicate that father's employment in a job with low psychological demand is associated with elevated odds for completed suicides among male children (Table 6).

## Discussion

Four main results arise from this study. First, even after partially controlling for the impact of a father's own history of mental health, male children of fathers with low duration of employment at a study sawmill while their child or children were less than age 16 had a greater odds of attempting suicide than children of fathers with high duration of employment. Second, female children of fathers who experienced low job control during the first 16 years of their child's life had significantly greater odds for attempting suicide. Third, after partially controlling for father's history of mental illness and other potential confounders, male children of fathers employed in jobs with low psychological demand showed significantly greater odds for completing suicide.

These results indicate that adverse work conditions for fathers experienced while their children are growing up may have serious psychological outcomes for these children. This investigation also shows that the impact of fathers' work experience may differ for males and females in relation to attempted suicide. This is consistent with the literature which shows that economic downturns may impact mental health outcomes among children and that the impact may differ for boys and girls [15].

Most of the children in this investigation were resident in small rural resource dependent communities in British Columbia during their childhood. Given that resource dependent communities in British Columbia have been exposed to continuous and intensive restructuring and downsizing since the early 1980s, these children may have been particularly "at risk" for adverse mental health outcomes including suicide. Research from Australia, Norway, and the United Kingdom have found the largest increases in suicide rates for young adults since the 1970s occurred among residents in rural regions [36-41].

There are several limitations to this study. First, the literature indicates that an important mediating factor between low family social economic circumstances during childhood and future health status of the children may be father's parenting behaviour. However, much of the evidence (but not all) for this was obtained in studies dating from the 1930s and the 1980s when father's role as breadwinner was more prominent than at present. Today, with female labour force participation rates approaching those of males, it is much more important to understand both mothers and fathers employment circumstances in relation to their children's health.

We have no data on mother's employment circumstances in this study. This limitation may be ameliorated to some extent because the investigation was conducted both historically and in a labour market characterized by fairly "traditional" gender roles. Thus, father's employment conditions were likely, given the context of this particular study, to have in fact been more important than the mothers'.

Second, data on place of residence for children was not available. These analyses assume that children resided with their fathers while he was working at a study mill during his children's first 16 years of life. However, it is quite likely that divorces took place and some of these children were residing with their mothers and therefore under-exposed to adverse paternal work circumstances during their childhood. Given that the socio-economic range within this fairly homogenous group of sawmill workers was fairly narrow it is unlikely that divorce rates differed systematically for cases and controls.

Third, we were able to link 88 percent of the children to the BCLHDB. Most of the 12 percent of children in the cohort who we were unable to link to the BCLHDB were likely younger healthy children who had not yet had contact with hospitals or physicians. Given that very young children are unlikely to attempt or commit children this will not bias our study. However, to the extent that we did not link to older children, because they were healthy enough to have avoided the healthcare system and therefore not be present in the BCLHDB, this could have introduced bias to slightly strengthen the observed associations in this study. Given the relatively high probabilistic linkage efficiency in this study this is likely to be of minimal concern.

Fourth, the study is based on cases admitted to acute-care and to psychiatric hospitals across BC. It does not record admissions for attempted suicide at community treatment facilities. As well, some people who attempted suicide may not present to physicians and may not even acknowledge their attempts to friends and family so that some unknown number of attempted suicides will have been missed in this study. However, given that the mental health care system in the province is heavily weighted towards hospital care, it is likely that only a very few attempted suicides would have been missed.

Fifth, the average age of men in the fathers cohort would have been about 50 years in 1985, the date when information on their mental health outcomes became available. This means that suicide and mental health outcomes that occurred when these men were younger, prior to 1985, were not recorded. This means that our statistical adjustment for father's suicides, alcohol-related mental health and other mental health outcomes are only partial. Full adjustment for father's mental health status (i.e., adjustment taking account of mental health events going back to father's childhoods) may slightly attenuate the observed associations between psychosocial work conditions and their children's suicide outcomes.

A strength of this investigation arises because of the complex data sets developed including the ability to control, at least partially, for the impact of father's history of mental health prior to an attempted or completed suicide by their child. In order to move these kinds of trans-generational analyses forward, unusual data sets of this type are required.

Furthermore, a full history of employment for fathers would have been ideal for use in this study. We only had father's employment while employed in a study sawmill. But, we applied fathers sawmill employment exposure to a specific window of time during their children's lives (i.e., during their first 16 years). Thus, we have utilized the data in a way that is consistent with the now well developed models of the importance of exposure to adverse socio-economic conditions early in life. This too is a strength of the analytical approach taken in this investigation.

## Conclusion

Using a unique data set, this investigation builds upon an older literature that has linked paternal exposure to adverse employment conditions during the early stages of their children's lives to adverse mental health outcomes among these children. While this earlier literature focused mainly on paternal exposure to unemployment this investigation has also demonstrated that prolonged exposure of children to adverse paternal psychosocial work conditions may also be implicated in suicide outcomes for these children.

## Abbreviations

BC = British Columbia

BCLHDB = British Columbia Linked Health Database

ICD = International Classification of Disease

OR = Odds Ratio

CI = 95% Confidence Interval

## Competing interests

The author(s) declare that they have no competing interests.

## Authors' contributions

ASO guided the analyses, developed the methods, and drafted the paper. JT helped guide the analysis and aided in writing the paper. SM guided the analysis and aided in writing the paper. JD aided in the analysis RH conducted the analysis. LC conducted the analysis. AML conducted and wrote the literature review. CH guided the analysis, and developed the methods. All authors read and approved the final manuscript.

## Pre-publication history

The pre-publication history for this paper can be accessed here:



## Figures and Tables

**Table 1:** Attempted and completed suicides by gender among members of the children's cohort from 1985 to 2001

**Suicides**	**Male**	**Female**	**Unknown gender**	**Total**
**Completed**	20 (77%) *	4 (15%)	2 (8%)	26 (100)
**Attempted**	81 (35.8%)	145 (64.2%)		226 (100)
**Total**	101 (40.1)	149 (59.1)	2 (0.8)	252 (100)

* Numbers in parentheses are row percents

**Table 2:** Socio-demographic and occupational characteristics of the fathers of case and control children

**Independent Variables**	**Attempted Suicides**		**Completed Suicides**	
	**Cases**	**Controls**	**Cases**	**Controls**

**Father's Marital Status**				
% Unmarried (N)	13.9 (29)	8.0 (50)	12.5 (3)	8.0 (6)
% Married (N)	86.1 (179)	92.0 (572)	87.5 (21)	92.0 (69)
**Father's Ethnicity**				
% Caucasian (N)	88.1 (199)	85.3 (578)	88,5 (23)	69.2 (54)
% Sikh (N)	10.6 (24)	13.3 (90)	11.5 (3)	25.6 (20)
% Chinese or Other Asian (N)	1.3 (3)	1.5 (10)	0	5.1 (4)
**Father's Occupational Status**				
% Manager (N)	6.2 (14)	6.9 (47)	3.8 (1)	5.1 (4)
% Tradesman (N)	30.1 (68)	34.5 (234)	26.9 (7)	39.7 (31)
% Skilled (N)	17.7 (40)	19.8 (134)	30.8 (8)	19.2 (15)
% Unskilled (N)	46.0 (104)	38.8 (263)	38.5 (10)	35.9 (28)
**Father's average duration (years) of employment at sawmill **(sd)	5.7 (4.1)	6.8 (4.1)	5.8 (3.9)	7.9 (3.9)
**Father's average # of episodes of unemployment **(sd)	1.05 (1.2)	1.12 (1.3)	1.08 (1.23)	1.14 (1.34)

**Table 3:** Univariate models for attempted suicides among children of sawmill workers during the first 16 years of the child's life.

**Independent Variables**	**Males**	**Females**
**Father's ethnicity (ref = Caucasian)**		
Sikh	.42 (.16,1.12)* (.08)**	1.0 (.58,1.73) (1.0)
Chinese	1.0 (.10,.88,1.14) (.95)	.85 (.17,4.31) (.84)
**Father's marital status (ref = married)**	1.00 (.88,1.14) (.95)	.97 (.88,1.07) (.52)
Father's suicide attempt ***	3.0 (.61,14.86) (.18)	**-**
Father's mental health diagnosis	1.42 (.79,2.57) (.24)	**1.97 (1.19,3.25) (.01)**
Father's alcohol diagnosis	**3.75 (1.00,13.96) (.05)**	1.5 (.45,4.98) (.51)
**Father's Occupational Status (ref = manger)**		
Tradesman	**.56 (.32,1.0) (.05)**	.99 (.66,1.49) (.96)
Skilled	1.0 (.52,1.91) (1.0)	.80 (.49,1.32) (.39)
Unskilled	**1.84 (1.08,3.14) (.03)**	1.17 (.79,1.73) (.43)
Father's average duration (years) of employment at sawmill	**.92 (.86,.97) (.00)**	**.95 (.91,.997) (.04)**
Father's average # of episodes of unemployment	.92 (.75,1.13) (.42)	.97 (.84,1.13) (.72)
**Psychosocial and physical work conditions**		
Control	.94 (.86,1.02) (.14)	**.92 (.86,.99) (.03)**
Psychological demand	.91 (.78,1.07) (.27)	.99 (.88,1.12) (.86)
Physical demand	1.55 (.86,2.80) (.14)	***1.60 (1.00,2.57) (.05)***
Social support	.88 (.59,1.3) (.52)	.95 (.70,1.29) (.75)
Noise	.80 (.46,1.39) (.43)	1.02 (.62,1.68) (.93)

* 95% Confidence intervals; ** p-value; ***data are missing as no father attempted or completed suicide prior to their female child's suicide attempt.

**Table 4:** Multivariate models for attempted suicides among *male *children of sawmill workers during the first 16 years of the child's life

**Independent Variables**	**Model 1**	**Model 2**	**Model 3**
**Father's ethnicity (ref = Caucasian)**			
Sikh	.55 (.20,1.53)* (.25)**	.58 (.21,1.63) (.30)	.52 (.18,1.49) (.22)
Chinese	1.05 (.11,10.32) (.96)	1.09 (.11,10.7) (.94)	1.12 (.11,11.47) (.92)
**Father's marital status (ref = married)**	.98 (.86,1.11) (.73)	.96 (.85,1.1) (.59)	.95 (.83,1.08) (.44)
Father's suicide		1.98 (.36,10.93) (.43)	1.75 (.31,9.94) (.53)
Father's mental health diagnosis		1.26 (.65,2.45) (.50)	1.26 (.65,2.47) (.49)
Father's alcohol diagnosis		3.58 (.87,14.71) (.08)	3.68 (.87,15.50) (.08)
**Father's Occupational Status (ref = manger)**			
Tradesman			1.03 (.29,3.69) (.96)
Skilled			1.48 (.41,5.36) (.55)
Unskilled			1.83 (.55,6.12) (.33)
Father's average duration (years) of employment at sawmill	**.92 (.87,.98) (.01)**	**.92 (.86,.98) (.01)**	**.93 (.87,1.00) (.05)**

* 95% Confidence intervals; ** p-value.

**Table 5:** Multivariate models for attempted suicides among *female *children of sawmill workers during the first 16 years of the child's life

**Independent Variables**	**Model 1**	**Model 2**	**Model 3**
**Father's ethnicity (ref = Caucasian)**			
Sikh	.86 (.49,1.54)* (.63)**	.73 (.14,3.77) (.70)	.76 (.15,3.96) (.74)
Chinese	.85 (.13,3.33) (.61)	.73 (.14,3.77) (.70)	.76 (.15,3.96) (.74)
**Father's marital status (ref = married)**	.96 (.87,1.06) (.43)	.97 (.87,1.07) (.52)	.96 (.87,1.07)(.49)
**Father's Occupational Status (ref = manger)**			
Tradesman			.76 (.31,1.85) (.54)
Skilled			.53 (.20,1.40) (.20)
Unskilled			.45 (.16,1.22) (.12)
Father's average duration (years) of employment at sawmill	.96 (.91,1.01) (.09)	.96 (.91,1.01) (.10)	.95 (.90,1.00) (.06)
**Psychosocial and physical work conditions**			
Control	.95 (.88,1.02) (.15)	.95 (.88,1.02) (.20)	**.90 (.81,.99) (.04)**
Physical	1.31 (.77,2.22) (.32)	1.37 (.80,2.36) (.25)	1.51 (.82,2.76) (.18)
Father's suicide ***		-	-
Father's mental health diagnosis		**1.95 (1.13,3.37) (.02)**	**2.01 (1.16,3.49) (.01)**
Father's alcohol diagnosis		.93 (.22,3.87) (.92)	.82 (.19,3.50) (.79)

* 95% Confidence intervals; ** p-value; ***data are missing as no father of a female child who attempted suicide had attempted or completed suicide prior to their child's attempt.

**Table 6:** Multivariate model for completed suicides among *male *children of sawmill workers during the first 16 years of the child's life

**Independent Variables**	**Model 1**	**Model 2**	**Model 3**
**Father's ethnicity (ref = Caucasian)**			
Sikh	.37 (.04,3.37)* (.38)**	.33 (.04,3.08) (.33)	.36 (.03,4.56) (.43)
Chinese***	-	-	-
**Father's marital status (ref = married)**	1.13 (.78,1.61) (.52)	1.13 (.76,1.67) (.56)	1.23 (.76,1.98) (.40)
Father's suicide	-	-	-
Father's mental health diagnosis	.11 (.01,1.42) (.09	.12 (.01,1.42) (.09)	.13 (.01,1.47) (.10)
Father's alcohol diagnosis ****	-	-	-
**Father's Occupational Status (ref = manger)**			
Tradesman			.82 (.05,13.78) (.89)
Skilled			3.14 (.17,57.56) (.44)
Father's average duration (years) of employment at sawmill	.98 (.86,1.13) (.82)	.99 (.86,1.14) (.89))	.97 (.80,1.16) (.71)
**Psychosocial and physical work conditions**			
Psychological demand	**.53 (.30,.95) (.03)**	**.40 (.18,.90) (.03)**	**.42 (.20,.98) (.04)**

* 95% Confidence intervals; ** p-value; ***data are missing as no Chinese fathers had a male child who completed suicide; ****data are missing as no father of a case or control had an alcohol-related mental health diagnosis.
